# Le cholédococèle: une variété rare de dilatation kystique congénitale des voies biliaires

**DOI:** 10.11604/pamj.2018.29.156.12084

**Published:** 2018-03-16

**Authors:** Nabil Boudjenan Serradj, Benali Tabeti, Anisse Tidjane, Noureddine Benmaarouf

**Affiliations:** 1 Service de Chirurgie Hépatobiliaire et Greffe du Foie, Etablissement Hospitalo-Universitaire d’Oran, Algérie

**Keywords:** Cholédococèle, dilatation, kyste, Todani, Choledochocele, dilation, cyst, Todani

## Abstract

La dilatation kystique des voies biliaires (DKVB) est une pathologie rare, elle touche principalement les jeunes femmes, avec la douleur comme maitre symptôme. Son principal risque est la cancérisation. L'exérèse chirurgicale complète demeure le traitement de choix. La classification de TODANI établi cinq groupes de dilatations kystiques congénitales des voies biliaires. Le cholédococèle représente le type III et se définit comme une dilatation kystique isolée de l'ampoule de Vater. La rareté de cette variété de DKVB et la multitude de présentations cliniques de cette pathologie avaient conduit à un nombre réduit de publications dans la littérature médicale, et l'absence de référence concernant la prise en charge thérapeutique entre traitement endoscopique en plein progrès ,mais conservateur et un traitement chirurgical radical mais difficile à réaliser. Notre présentation porte sur le cas d'une jeune patiente âgée de 32 ans, consultant pour des douleurs épigastriques itératives et dont les examens morpho-cliniques conclut à un cholédococèle .Nous avions réalisé chez cette patiente une résection complète du kyste à travers une duodénotomie, avec réimplantation du canal cholédoque et du canal de Wirsung, associée à une cholécystectomie.

## Abstract (en)

*Cystic dilation of the bile ducts (CDBD) is a rare disease in which pain is the major symptom. It mainly affects young women. Carcinogenesis is the main risk of this disease. Complete surgical resection is the treatment of choice. TODANI's classification defines five groups of congenital cystic dilatation of the bile ducts. Choledochocele is classi?ed as type III and is defined as an isolated cystic dilation of the ampulla of Vater. Few studies were published in the literature due to its rarity and to its multitude of clinical presentations. The lack of guidelines resulted in approaches to treatment based on endoscopic treatment, making rapid progress but considered as a conservative option, or on hardly obtainable complete surgical resection. This study reports the case of a 32-year old female patient presenting with cyclic epigastric pain. Morpho-clinical examinations showed holedochocele. Complete resection of the cyst via duodenotomy with reimplantation of the common bile duct and of the Wirsung duct associated with cholecystectomy were performed.*

## Introduction

La dilatation kystique des voies biliaires (DKVB) représente l'ensemble des malformations congénitales qui se caractérisent par une ou plusieurs dilatations kystiques communicantes des voies biliaires. Représentant une variété rare au sein même de ces DKVB, le cholédococèle reste une entité clinique, iconographique et thérapeutique peu connue des praticiens.

## Patient et observation

**Figure 1:**
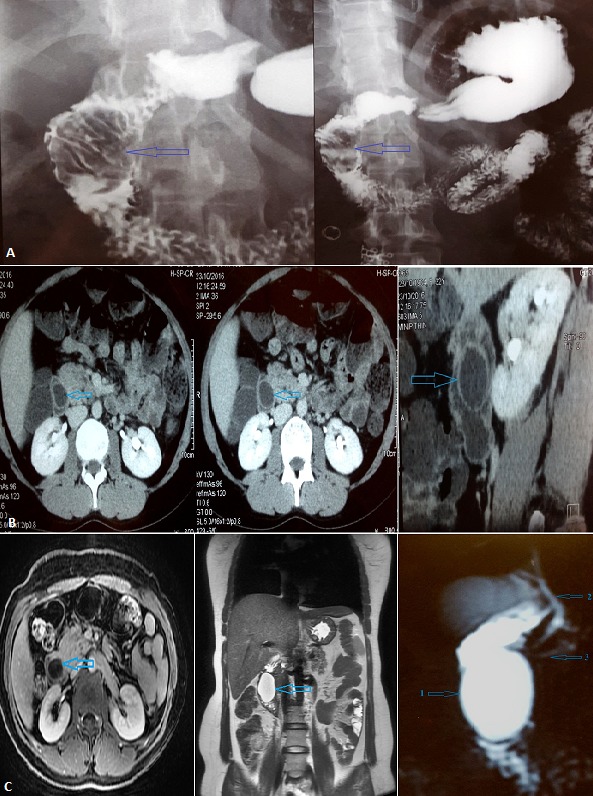
Imagerie préopératoire: A) transitoeso-gastro-duodénal ; B) scanner abdominal ; C) imagerie par résonance magnétique : 1) le cholédococèle ;2) canal cholédoque ; 3) canal de Wirsung

**Figure 2:**
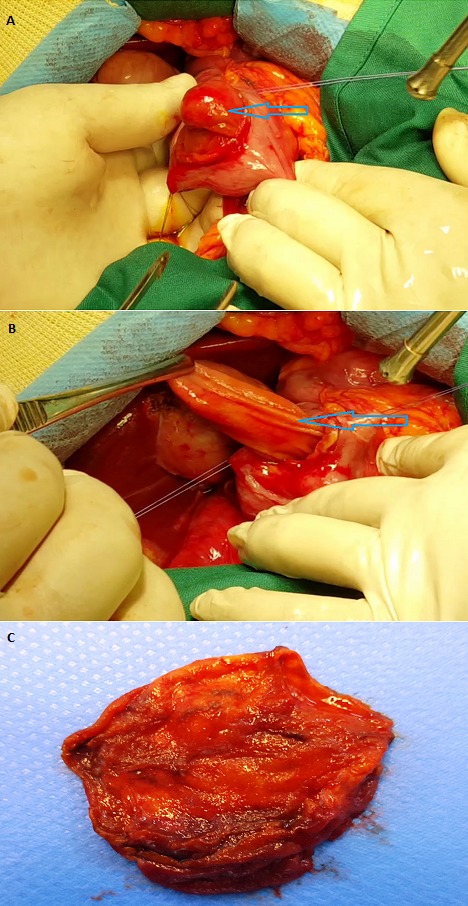
Vue per opératoire : A et B) flèche bleue : le cholédococèle; C) pièce de résection

Nous rapportons le cas d'une jeune patiente âgée de 32 ans, sans antécédents particuliers, consultant pour des douleurs épigastriques itératives. Une série d'examens (Figure 1) a été réalisé concluant à une formation kystique endoluminale au dépends du 2^ème^ duodénum avec des rapports étroits avec le cholédoque terminal et le canal de Wirsung. L'exploration chirurgicale, à travers une duodénotomie concluait à une dilatation kystique de l'ampoule de Vater centrée sur la papille, réalisant une dilatation kystique congénitale des voies biliaires type III de Todani ou cholédococèle [1-3] (Figure 2). Nous avions réalisé une résection complète du kyste, avec réimplantation du cholédoque et du canal de Wirsung associée à une cholécystectomie, avec des suites opératoires simples et disparition complète de la symptomatologie initiale. L'examen anatomopathologique de la pièce d'exérèse concluait à une dilatation kystique de l'ampoule de Vater sans signes de dysplasie ou de transformation maligne.

## Discussion

La dilatation kystique des voies biliaires est une pathologie congénitale rare. Son incidence est beaucoup plus élevée en Asie du sud est et au Japon, cependant une fréquence de l'ordre 20 à 50% des cas sont découverts à l'âge adulte, avec nette prédominance féminine [4-6]. Le principal risque évolutif des DKVB demeure la dégénérescence, car on estime que l'incidence du cancer des voies biliaires en cas de DKCVB est se situe entre 3 à 40% [7,8]. Elle augmente avec l'âge et en cas de dérivation kysto-digestive [9,10]. Le cholédococèle représente une dilatation kystique isolée de l'ampoule de Vater et correspond au type III de la classification de Todani [3]. Il représente 2 à 4% de l'ensemble des dilatations kystiques des voies biliaires [1]. Malgré un faible taux de dégénérescence du cholédococèle décrit dans les rares cas publiés dans la littérature, celui-ci reste toujours un facteur de risque de cancer des voies biliaires [11]. Le traitement du cholédococèle peut être endoscopique ou chirurgical. Le principe de ce dernier est l'exérèse de la totalité du kyste, conduisant à une suppression du reflux wirsungo-biliaire responsable de la transformation maligne de la dilatation kystique [1]. Le traitement chirurgical consiste à une exérèse trans- duodénale associée à une sphinctéroplastie chirurgicale [1,3,12]. Celui-ci est associé à une morbidité faible et mortalité presque nulle dans les mains d'équipes spécialisées, rendant le traitement chirurgical une valeur sure dans la prise en charge thérapeutique des dilatations kystiques congénitales des voies biliaires en général et du cholédococèle en particulier.

## Conclusion

Le traitement chirurgical, de part la résection complète de la dilatation kystique, de la suppression du reflux pancréatique dans les voies biliaires et la faible morbi-mortalité de l'acte chirurgical dans les mains d'équipes spécialisées, demeure une alternative sure dans l'arsenal thérapeutique de la prise en charge du cholédococèle. L'absence de séries importantes dans la littérature mondiale rend difficile l'établissement de références ou de standards concernant la prise en charge thérapeutique du cholédococèle.

## Conflits d’intérêts

Les auteurs ne déclarent aucun conflit d'intérêts.
